# Trichina Spiralis and Trichiniasis

**Published:** 1875-02

**Authors:** Geo. E. Blackham

**Affiliations:** Dunkirk, N. Y.


BUFFALO

### Medical and Surgical Journal.

VOL XIV. FEBRUARY, 1875. No. 7.

### Original Communications.

———:o:———

ART. I.—*Trichina Spiralis and Trichiniasis. Read before the Chautauqua County Medical Society.* By GEO. E. BLACKHAM, M. D., Dunkirk, N. Y.

Published by request of the Society.

Having lately had occasion to examine some meat suspected of causing *trichiniases* in two families, one here and one in Buffalo, and having in both instances been able to verify the diagnosis by demonstrating the presence of the parasite in the suspected meat. I have, at the request of the president of the Society, brought with me my microscope and some specimens of the trichinatous meat, both in bulk and mounted, and have prepared a sketch of the disease with more particular accounts of the late cases in Buffalo and Dunkirk.

Of all known parasites which infest the human body the *Trichina Spiralis* is at once the most minute and the most deadly. Though there can be little doubt that they have existed from the earliest times, yet it is scarcely more than fifty years since their existence has been recognized, and less than fifteen since their dangerous character has been actually known, and the disease to which they give rise has been recognized and described. Yet it is probably of more frequent occurrence than has been supposed.

Zenker of Dresden, having found *trichinæ* in four out of one hundred and thirty-six dissections—about one in forty-four.

The following is a brief review of its medical history:

So far as I can discover it was first seen by Tiedemann in 1822, who, however, believed it harmless and not worthy even of a name. In 1832, Mr. Hilton of Guys Hospital, London, called attention to the same parasite in human voluntary muscle. In 1835, Mr. Wormald of St. Bartholomew's Hospital sent to Prof. Owen a piece of human muscle which presented a peculiar appearance, being dotted over with minute whitish specks which were described as looking like mould spots. On examination with the microscope, Prof. Owen found each mould spot to be a shuttle shaped cryst containing from one to three minute hair-like worms coiled spirally. From these two characteristics he gave it the name *Trichina Spiralis*, by which it has since been known.

Mr. Paget, while an under graduate, read a paper on this parasite a week prior to Mr. Owen's, but did not suggest a name. From 1835 to 1860, the *trichina* was recognized occasionally in the dissecting room, but little or no importance was attached to it, as it was supposed to be harmless. In 1860, however, Prof. Zencker of Dresden, had his attention called to the case of a young girl who died in the Dresden Hospital about the 28th or 30th day of an illness which had been set down as typhoid fever, though not without grave doubts as to the correctness of the diagnosis. Her illness had begun about Christmas, 1859. A post mortem examination showed the muscles moderately developed, of a pale reddish gray color, and dotted with specks which proved to be groups of free *trichinæ* lying upon and within the sheaths of the muscular fibres. They were alive, some were coiled and others straight. They appeared in all stages of development, and were found in all the striated muscles not excepting those of the heart. They were exceedingly numerous, as many as twenty being seen in a single field of the microscope when a low power was employed. The muscular tissue everywhere showed a marked degree of degeneration.

On looking into the history of the girl, she was found to have been taken ill soon after eating of the flesh of two pigs which had been killed about Christmas. Prof. Zencker went to her master's, and found some of the flesh of the pigs remaining, and also some sausages. The flesh of the pigs, on being examined microscopically, was found to be infested with *trichinæ* in the encysted state. Other servants in the same house, and also the butcher who had killed the pigs, were found to have been taken sick about the same time with symptoms varying principally as to severity. Numerous experiments were made with the flesh of this girl. Some was sent to Virchow at Berlin, who fed some of it to rabbits which died in about a month with symptoms of general muscular paralysis, and on examination their flesh was found to be swarming with *trichinæ*.

In the summer of 1860, a trichinatous subject was received into the dissecting room of the University of Edinburgh, and the Demonstrator of Anatomy, Dr. Turner, repeated and verified the experiments of Virchow.

During an operation on the neck of a patient in 1863, Prof. Langenbeck noticed a peculiar appearance of the *platysma myoides* which proved to be due to the presence of *trichinæ* in cysts which had become cretified. On enquiry the following facts were elicited: In 1845, seven person sat down to a breakfast of ham, sausages, cheese, roast veal and white wine. In the course of a few days every one of these persons was taken with diarrhœa, pains in the neck, and œdema of the face and extremities. Four died, and the three who survived (Langenbeck's patient being one) were sick for a long time. The inn-keeper at whose house they had eaten breakfast was suspected of having poisoned the wine, his business was broken up and he was obliged to emigrate. This suggests that some cases of suspected poisoning may be due to this parasite.

In October, 1863, on some festive occasion in Heldstadt, in Prussia, 103 persons sat down to an apparently excellent dinner, and of this number 100 were soon attacked with symptoms of *Trichiniasis* and forty died. *Trichinæ* were found in abundance in the remnants of the pork and sausages which had formed a portion of the dinner.

In 1865, there was an outbreak of this disease at Hedersleben in which 300 persons were attacked and 40 died.

The first cases in the United States were reported by Dr. Joseph

Schnetter of New York. Several members of a family were taken sick soon after eating raw smoked ham. Dr. Schnetter and Prof. John C. Dalton examined portions of the ham and found them so filled with *trichinæ* that Prof. Dalton estimated it to contain about 85,000 to the cubic inch of lean meat. Soon after, Dr. Voss of New York had four cases on board one of the Bremen steamers. He verified his diagnosis by cutting down on and removing a portion of the deltoid muscle of one of his patients, and finding it to be filled with *trichinæ*. The next cases occurred in the practice of Drs. Krombein and Dingler, German physicians of Erie County, N. Y., and were reported in the BUFFALO MEDICAL AND SURGICAL JOURNAL, by Dr. J. R. Lothrop. In 1866, fifteen cases occurred in Marion, Iowa, nine of which were reported by Dr. J. H. Wilson, and six by Dr. H. Ristine. About a year ago several cases occurred in a family in Buffalo, N. Y., of which I have not the particulars. The next cases, of which I have found a record, occurred in Detroit in 1874, and are reported by Dr. Judson Bradley, in an excellent article published in the Detroit Review of Medicine and Pharmacy for January, 1875. The latest cases occurred in Buffalo and Dunkirk within the last month, and are those in which I had the opportunity of verifying the diagnosis by finding *trichinæ* in some remnants of the sausages which had caused the disease.

The following brief clinical histories of these cases were kindly furnished to me by Dr. E. N. Brush, editor of the BUFFALO MEDICAL AND SURGICAL JOURNAL, who saw the Buffalo cases, and Dr. H. R. Rogers of Dunkirk, who has had charge of the Dunkirk cases up to the present time.

The history of the Buffalo cases is as follows: I quote Dr. Brush's letter—"A butcher in the Clinton Street Market, Buffalo, has been in the habit of making what are called 'Smoked Pork Sausage,' in quantities of from 200 to 300 pounds. Dec. 5, 1874, Mrs. Miller, residing at 352 William st., Buffalo, ate in the market a portion of this sausage. She had, however, eaten a small piece at the house of her mother, Mrs. Leith, about a week previous. On Sunday and Monday, December 6th and 7th, she was sick with diarrhœa and vomiting with some abdominal tenderness. She did not pay much attention to the illness and could not, at the time, assign a cause for it. No other member of the family had eaten of the sausage, and no one else was sick. When I saw her on the evening of Dec. 18th, she looked pale and anxious, was bent over and could not stand erect without great pain. There was pain in the calves of the legs, in the arm and in the pectoral muscles.

Mrs. Leith, aged 57, mother of Mrs. Miller, resides with her son at 191 Hickory st., purchased on Saturday, Nov. 28th, some sausage from the maker in the Clinton st., Market, (the same who sold to Mrs. Miller). She ate of the sausage as did also her sons, Lewis, aged 26, and William, aged 19, who reside with her. She gave a portion to her daughter, Mrs. Fisher, who resides up stairs. All these persons were taken sick in periods varying from 48 to 72 hours from the time the sausage were first eaten. Other members of these three families who did not eat of the sausage are in good health. The symptoms first observed were pain, somewhat resembling colic, diarrhœa, which, in one or two cases, was profuse, and vomiting. In the course of a week or ten days, muscular pain was observed, which, in character, resembled that of inflammatory rheumatism. One case resembled, from what I can learn, for the first week, a mild case of typhoid fever. In all there was profuse perspiration at times. In the case of Lewis Leith an eruption appeared, which, taken in connection with slight conjunctivitis and œdema of the lower lids, closely resembled that produced in some persons by the administration of large doses of potass. iodid. This œdema, which is said to be a characteristic symptom, was more noticeable in the morning on arising, was present in all, and in one case was very marked indeed.

The present condition of the five cases (Jan. 7, 1875,) is as follows:

Mrs. Leith (the mother) is able to be about her house work, has some diarrhœa and slight muscular pains in arms and legs, is weak but otherwise well.

Mrs. Miller is very sick, has diarrhœa, severe pain, sweats, and is greatly prostrated. She is confined to her bed, but is very restless, and tries every conceivable position to obtain ease.

Mrs. Fisher is nearly well, has some diarrhœa and abdominal pain, also slight pains in arms and in calves of legs.

Wm. Leith has but very little pain and occasional diarrhœa; the

œdema of the eyelids still continues. He is well enough to engage in his business.

The cases have all been under the professional care of Dr. Tobie."

The following extract from a letter received from Dr. H. R. Rogers, dated January 7th, gives concisely the history of the Dunkirk cases up to that date:

"I send you, according to your request, a concise history of the Eiselben family of whom nine of its ten members were subjects of *Trichina Spiralis*, (the tenth was an infant).

The family partook of the uncooked meat about the 13th of December. The first indication of disturbance as probably resulting from that source occurred to Mrs. E. about the 20th, and to the remainder of the family on that or the following day. The first symptoms were uneasiness of the stomach and bowels and diarrhœa. About the 25th, Mrs. E. consulted me at my office, was informed of the character of the disease, and received medicine suited to the case. On the 28th I was called to visit the family, and found Mrs. E., a son about seven years old, and a daughter about two years old, vomiting severely. This confirmed my diagnosis, and treatment was immediately followed by good results. Since the 29th, the family have been comparatively well. To day I again visited them for the first time since the 29th, for the purpose of reporting results to you, and found some disturbance of the bowels yet continuing. A son of ten years has some pains in the region of the false ribs, and his countenance appears quite bleached. This latter condition I observed in the cases of several others of the family. Six of the children are attending school, and the father and mother are attending to their usual duties.

The treatment may interest you. To Mrs. E., at my office, I gave active cathartics for the whole family, and an anodyne diarrhœa mixture. This course had the effect to improve the condition of all until the 28th, at which date I found the condition before mentioned. For the vomiting, as no remedy would remain for a moment upon the stomach, I used for the wife and son 1-6 grain doses of acetate of morphia, hypodermically, with directions to take nothing into the mouth but pounded ice, that *ad libitum*.

On the 29th, large doses of castor oil and ol. turpentine were ordered for each patient, since which time the family have not consulted me, having regarded themselves as very well. They were, however, told the necessity of continued and active treatment, but chose to regard the opinions of neighbors who, from the unusual mildness of the cases, supposed that perhaps a mistake had been made in the diagnosis.

The wife, to-day, knowing that they have acted unwisely, has requested me to resume my services, and I shall take pleasure in keeping you informed as to the results."

So much for the history of the disease, from the discovery of the parasite, in 1822, to its recognition as a cause of disease, in 1860, and from that to the particulars of the very latest cases up to date. The following is the life history of the parasite itself. The *Trichina Spiralis* is a minute bisexual, viviparous worm, which in its earliest and latest stages inhabits the alimentary canal of various warm-blooded animals, and passes an intermediate stage imbedded in the voluntary muscles. When meat containing the immature *trichinæ* is eaten by man or any other animal, the cysts are dissolved by the acids of the stomach, and the *trichinæ* set free. They grow rapidly, loose their spiral figure, their generative organs are developed, and in two days they attain their full sexual maturity. At this time the males are about one-eighteenth, and the females about one-eighth of an inch in length, and the latter contain from 200 to 500 ova each. These ova are developed within the mother into minute embryos, which on the sixth or seventh day are born without their egg shells. The new-born young soon begin their migrations; penetrating the walls of the intestines they make their way into the voluntary muscles in every part of the body, penetrating into the interior of the separate muscular bundles where, after fourteen days, they assume the form in which they are best known, and are enclosed in their peculiar cysts which are formed thus: Soon after the entrance of the parasite the muscular fribrillae undergo a sort of granular degeneration, and the muscular corpuscules change into oval, nucleated cells. The original sheath of the bundle remains intact till the young trichinae have attained their complete development; soon afterward, however, the sarcolemma begins to thicken and shrivel at the extremities, enclosing the coiled-up parasite in a spindle or shuttle-shaped space, within which the cysts are formed by the peripheric deposition of calcareous matter, (carbonate of lime), at first transparent but afterwards becoming, in the course of a long time, cretaceous and visible to the naked eye as the minute whitish spots first described by Prof. Owen. Here the parasites remain, living but quiescent, till the flesh of their victim is eaten by some other animal, when the same process is repeated.

The disorder produced by them may be divided into three stages: First, from the ingestion of the immature *trichinæ* till the young are born, symptoms—general malaise, gastric trouble, diarrhœa, and vomiting. Second, from the beginning of their migration till they are encysted in the muscles, symptoms—fever, profuse perspirations, blanching and oedema of the face, (sometimes general dropsy,) conjunctivitis, photophobia, scanty, high-colored urine, diarrhœa and general tenderness of the abdomen, restlessness, formication; severe muscular pains, first in the back and neck, next in the arms and thighs, and lastly in the forearms and calves. Hiccough and dyspnœa result from the invasion of the respiratory muscles, hoarseness from the invasion of those of the larynx, etc. The decubitus is dorsal with the legs drawn up. The fever continues, typhoid symptoms set in, with meteorism, restlessness, and sometimes delirium. Pneumonia may occur with pleural effusion. Death may end this stage in from two to six days, but it ordinarily lasts from three to five, or six weeks. Attacks are sometimes very insidious; a patient who has not been severely ill, may die suddenly from pneumonia or peritonitis. Third, the stage of recovery. The symptoms lessen on the re-appearance of the urinary secretion, but muscular stiffness may last for some time, and baldness and epidermic desquamation may take place.

This is the course in severe cases, but similar symptoms may be observed in all degrees of severity in milder cases.

The principal difficulty in diagnosis will be the differentiation of *Trichiniasis* from *typhoid fever*. The following are the distinctive points: The enlargement of the spleen, expistaxis, gurgling on pressure in the right iliac region, and the rose spots of typhoid fever are all wanting in trichiniasis; while characteristic disturbance of the digestive organs, followed by oedema of the face, severe muscular pain, especially on motion, with breathlessness, often threatening asphyxia, present a series of regular and connected symptoms corresponding to the ingestion, reproduction and migration of the parasites, which occurs in no other disorder. The diagnosis may be made absolute by the discovery of *trichinæ* in meat of which the patient has eaten a portion, or in a portion taken from the muscles of the patient himself, which may be done almost painlessly with one of Duchenne's harpoons. The latter procedure, however, is seldom necessary and could be of little use as a guide for treatment, as it would probably give only negative results prior to the fourteenth day, when most of the *trichinæ* would be safely imbedded in the muscles out of the reach of any vermicide drugs. Treatment to be of any use must begin within a week after the ingestion of the immature *trichinæ.* In that stage, emetics and cathartics may remove the worms, or a large proportion of them from the intestinal canal, but after the new brood have begun their wanderings they are out of the reach of medicine. Reliance must then be placed in anodynes and hypnotics to quiet pain and restlessness, and easily digested nourishing food and tonics to support the patient through the period of general irritation till the parasites have become encysted in his muscles. Of course, the occasional administration of a cathartic such as ol ricini et terebinthinæ in liberal doses, to remove any *trichiniæ* which may have remained lodged in the intestinal mucous, would be rational and judicious. Various remedies for the destruction of the worm have been proposed, and have uniformly failed. Picric acid was proposed, but in the flesh of a pig killed with it, *trichiniæ* were found alive. Dr. Mosler suggests benzine, but can show no good results from its use. Dr. Tavernier suggests carbolic acid, and so on through a long list. All vermicides must fail, because the symptoms which suggest their use also show that the majority of the parasites are already out of their reach. Dr. Bradley, of Detroit, advocates the use of large doses of quinia sulph. and tinct. ferri chlorid. on the ground that of two patients under his care in the Detroit City Hospital, last winter, the wife who was so treated recovered, and the husband who was *not* so treated, died. But to my mind a more rational explanation of the death of one and the recovery of the other is to be found in the fact, stated by Dr. Bradley, that "The woman stated that she ate but little of the meat that caused the mischief in the man." *Hopes of recovery must mainly be founded on the smallness of the number of living trichinæ ingested, and the strength of the patient to endure the irritation.* Since then treatment can be of little avail, except in the very first stages of the disease, it is pertinent to inquire what means of prevention are available. They are, first, the *thorough cooking* of all pork. *All parts* of the meat should be subjected to a heat of *at least* 160° Farenheit before being eaten. If the inner portion of a piece of boiled or roast pork has much of the blood color of raw meat, it has not been subjected to a higher heat than 131° Far., and there is still danger, as the encysted *trichinæ* require a higher heat to destroy them. Hot smoking continued for a long time is said to destroy them, but cold smoking does not; neither does the ordinary process of salting. Simply *toasting* sausages does not kill the parasite, but the moderate heat which reaches the interior of the sausage rather stimulates the vitality of the *trichiniæ*. *Every* part of the meat should be subjected to a temperature of 200° Farenheit in order to secure safety. Second, microscopic inspection of the flesh of every pig killed, and the destruction by fire of such as are found trichinatous. The objections to this course are its expense, the difficulty of getting properly qualified examiners, and the extreme distaste of our people to any invasion of their fancied "rights" in the interests of sanitary science.

Although hog cholera has been supposed to be caused by the *trichinæ*, yet I do not know that the fact has been demonstrated by actual experiment. Cobbold mentions an instance where the flesh of a pig that appeared remarkably healthy, produced death. Depelch, in his report to the French government, says: "The butchered meat looks too well." Some difficulties attend the discovery of the parasite with the microscope, even though the meat under examination may be swarming with it. It is not sufficient to cut off a piece of the meat, place it in the field of the microscope, and focus upon it. It must be rendered very thin and transparent before the minute and delicate worms can be seen in it.

I, at first, failed to find any specimens in the sausage sent me by Dr. Brush. Yet, after proper preparation, I was able to see in one of the samples, which I present here to-day, no less than six trichinæ, coiled up in a single field of the microscope occupying a space about one-twentieth of an inch in diameter. The plan which I found most available was to place thin sections of the suspected meat in a dilute liquor potassæ (1 to 8 of water), and as soon as they assumed a pearly aspect, to place them with a little clear water in a compressorium, which I extemporized out of a couple of ordinary slides, held firmly together by a spring clothespin at each end. The action of the potassa softened the meat so that it was easily pressed out into an exceedingly thin and transparent layer in which, with a power of forty diameters or more, the *trichinæ* could be distinctly seen, sometimes moving within their cysts, but more often lying coiled up and perfectly quiescent. Taking the averages of a series of observations, I have estimated that the sausages sent from Buffalo contained about 15,000 to 20,000, and the specimen sent me by Dr. Rogers, from 6,000 to 15,000 trichinæ to the cubic inch. Of course, such an estimate can be only vaguely approximate, especially in the case of sausages which, being made from portions of several animals chopped up together, one piece may be found swarming with *trichinæ*, while those surrounding it may be entirely free.

If the liquor potassæ be too strong, or the specimens be left in it too long or be not thoroughly washed in pure water before mounting, the action of the alkali may go on and ruin the specimen, as I see it has done in some of the specimens I have with me. Specimens may be mounted in balsam or in glycerine, and it is advantageous to stain them slightly with aniline red, or some other transparent dye. I present a sketch of specimens of *trichinæ* encysted, and removed from the cysts. The drawing was made through the microscope from a specimen, put up by a professional preparer of microscopic objects. It is magnified about one hundred and twenty diameters.

In conclusion I have to acknowledge my indebtedness to Drs. E. N. Brush and H. R. Rogers for their kindness in furnishing me with details of their cases, and specimens of the infected meat; and to the works of Drs. Flint and Hartshorn, to the late article of Dr. Bradley, but more especially to the able article on this subject in Aitken's Practice, and the copious notes of the American editor, Dr. Clymer, for many of the facts embodied in the present article.

NOTE.—It should be mentioned that *trichinæ* have been found in the flesh of many animals other than man and swine. All carnivorous animals are liable to swallow them with their food; and they have been found in sheep and oxen, and other ruminants who probably got them by drinking water which had been contaminated by excretions of carniverous animals infected with the disease.

